# Completion and Factors Associated with Maternity Continuum of Care among Mothers Who Gave Birth in the Last One Year in Enemay District, Northwest Ethiopia

**DOI:** 10.1155/2020/7019676

**Published:** 2020-09-01

**Authors:** Anguach Shitie, Nega Assefa, Merga Dhressa, Tenagework Dilnessa

**Affiliations:** ^1^College of Medicine and Health Sciences, Wollo University, Ethiopia; ^2^College of Health and Medical Sciences, Haramaya University, Ethiopia

## Abstract

**Background:**

Ethiopia still suffers high levels of neonatal and maternal mortality, so the maternity continuum of care is a continuous framework for the delivery of maternal care from pregnancy to the postnatal period. Skilled care during pregnancy, childbirth, and the postpartum period is an important intervention in reducing maternal and neonatal morbidity and mortality. But in Ethiopia, there are limited studies on the completion of the maternity continuum of care, so this study will help to suggest interventions in order to reduce the dropout of the maternity continuum of care.

**Objective:**

To assess the completion of the maternity continuum of care and factors associated with it among mothers who gave birth in the last one year in Enemay District, Northwest Ethiopia. *Method and Materials*. A community-based cross-sectional study was conducted from February 25 to March 10, 2019, on 651 women who gave birth in the last one year. The data were collected by a face-to-face interview through pretested and structured questionnaires. Binary logistic regression was used to identify predictors of the completion of the maternity continuum of care. Variables with a *P* value < 0.05 in multivariable analysis were declared as statistically significant associated factors.

**Results:**

This study revealed that about 45% (95% CI: 40.9%, 48.8%) of respondents completed the continuum of care. Women with secondary education (AOR = 6, 95% CI: 2.26, 16.6), women whose occupation is farming (AOR = 0.18, 95%, CI: 0.1, 0.32), women who have autonomy in health care decision (AOR = 4, 95% CI: 2.26, 7.2), women who have exposure to media (AOR = 1.97, 95% CI: 1.2, 3.27), women with wanted pregnancies (AOR = 3.33, 95% CI: 1.87, 5.9), para five and above women (AOR = 2.85, 95% CI: 1.28, 6.3), and women whose husbands are employed (AOR = 4.97, 95% CI: 1.16, 21.2) were significantly associated with the completion of the maternity continuum of care. *Conclusions and Recommendation*. This study showed that less than half of the participants had achieved the continuum of care and education level, and both respondents and their husband's occupation, parity, autonomy in health care decision, exposure to the mass media, and wantedness of pregnancy were associated with the completion of the maternity continuum of care; therefore, working on enhancing the capacity of women's autonomy in health care and decision-making and preventing unintended pregnancy helps to improve the completion of the maternity continuum of care.

## 1. Introduction

### 1.1. Background

A continuum of care approach to maternal health is being defended as a key program strategy or a means to guarantee that women were given essential services during pregnancy, delivery, and the postpartum period [1, 2]. The health care services that a woman receives during the continuum of maternal health care (pregnancy, childbirth, and the immediate postnatal period) are important for the survival and well-being of both the mother and the child [3]. The services included are antenatal care for pregnancy-related health care check-ups and having a skilled birth attendant for delivery and postnatal care through the postpartum period for mothers and their newborns. Primarily, all pregnant women should have sufficient and high-quality antenatal care (ANC) during pregnancy [4].

According to the World Health Organization (WHO) 2015 estimation, approximately 303,000 maternal deaths occurred globally. Most of the deaths occur during labor, delivery, and the immediate postpartum period [5]. Ethiopia is one of the countries with the highest maternal mortality levels in the world, with an estimated 676 deaths per 100,000 live births in 2011 [6]. According to EDHS 2016, the maternal mortality rate in Ethiopia was 412 deaths per 100,000 live births [7].

The causes of thousands of deaths in women and their newborns are still preventable, since those causes are due to pregnancy- and childbirth-related complications; care provided during the continuum of maternal health service (pregnancy, childbirth, and postnatal period) by a skilled health care provider is the important intervention to save the life of both the mother and their infant, but many women especially in the country where the maternal mortality ratio is highest do not get all the services in the continuum of maternal health service (antenatal care, skilled delivery care, and postnatal care) [8].

The World Health Organization recommends a minimum of four antenatal care visits [9]. However, global estimates indicate that only half of all pregnant women receive this recommended amount of care. In Ethiopia, only 32% of Ethiopian women with live birth received at least four visits during the length of their pregnancy [7], which is below the global average (54%). The predominant underlying factors for the low coverage of antenatal care services include sociocultural and economic barriers, poor access to health services, and poor quality of antenatal care services [10]. Many mothers who attend the recommended number of antenatal care visits fail to use facility delivery and postnatal care services. The most common service women received is at least one ANC visit which is 43%. Utilization of professional-assisted delivery care and PNC is very low. We see that the proportion of women delivery by a skilled birth attendant is 11.5 and the proportion of women who received PNC within 24 hours is only 5% [11]. It has been stated that the reasons for low utilization of delivery and postnatal care service were the unpredictable onset of labor, making it difficult for women to travel long distances, lack of information, inadequate services, and cultural practices as well as some factors associated with the cost of delivery of care [12, 13].

Most studies in Ethiopia focused on the status of the use of one or two components of maternal health care separately and examined factors associated with the utilization of the services. Therefore, identification of the magnitude and the possible factors that determine the completion of the maternity continuum of care will help to suggest interventions in order to reduce the dropout of the maternity continuum of care.

## 2. Methods and Materials

### 2.1. Study Area and Study Period

The study was conducted in Enemay District. Enemay District is one of the districts in the East Gojjam Zone which is located 270 km away from Addis Ababa. Enemay District is bordered on the south by Dejen, on the west by Debay Telatgen, on the north by Enarj Enawga, and on the east by Shebel Berenta. The administrative center of this district is Bichena. It has a total of 36 kebeles from which 28 are rural kebeles and 8 are urban kebeles. It has 7 health centers, 35 health posts, and 1 hospital. The 2017 statistical figure of the Enemay District Health Office report showed that the antenatal coverage of the district is about 63% and the institutional delivery 58%. The study was conducted from February 25 to March 10, 2019.

### 2.2. Sampling Procedure

Enemay District has a total of 36 kebeles from which 28 are rural and 8 are urban kebeles. From the 28 rural kebeles, 8 kebeles were selected randomly, and also from the 8 urban kebeles, 3 kebeles were selected randomly. Totally, 11 kebeles were selected from the total thirty-six kebeles by a simple random sampling method. The number of women who delivered in each selected kebele was taken from the health extension worker registration book. Then, the sample was proportionally allocated for each selected kebele. Finally, the simple random sampling method was employed to select the women by using guidance.

### 2.3. Sample Size Determination

To determine the sample size, a single population proportion formula was used for the first objective (dependent variable) by considering the estimated proportion of completion of the maternity continuum of care (*P*) = 50.4% [14]. The margin of error *d* = 5% and 95% confidence interval are assumed (*Zα*/2 = 1.96) which gives 384. For the second objective, the StatCalc function of Epi Info version 7 software was used by using a cohort or cross-sectional sample size calculation technique from StatCalc and by considering the following assumptions: Adjested odds ratio of women with higher education, para 1-2, and rural residence 3.73, 1.97, and 0.46, respectively, with 95% confidence interval, 80% power, 1 ratio of unexposed to exposed and percent outcome in unexposed group for women with higher education, para 1-2 and rural residence 22.2% 16.1% and 64.5, respectively; sample size by StatCalc software become 84 for higher education, 414 para 1-2 and 214 for rural residence. Therefore, the maximum sample size is high for the second objective which is 414 taken as the final sample size. After adding a nonresponse rate of 5% and a design effect of 1.5 to increase power, the total sample size will be 414 + 20 = 434 and the design effect 1.5 = 434∗1.5 = 651.

### 2.4. Data Collection Tool, Quality Control, and Measurement

Structured and pretested questionnaires which are adapted by reviewing different literature were used to collect the data. The first tool was prepared in English, translated to Amharic, and then translated back to English by experts to check for consistency. For administering the interview, 11 diploma nurses and 11 guidance counselors were recruited. Four BSc degree nurses were also being used for supervising activities along with the principal investigator. In addition, the data collectors were trained in one day on the techniques of data collection and the purpose of the study. A pretest was done on 5% of the total study participants, and necessary adjustment was made. Data completeness and consistency were checked, cleaned, and compiled by the investigator on a daily basis.

### 2.5. Measurements

#### 2.5.1. Dependent Variables

Complete care for maternal health care services was the outcome variable of interest. The continuum of care was considered complete when the women received the following services at three levels:

- At least one antenatal care during pregnancy
- Childbirth aided by skilled birth attendants
- PNC for the mothers and their newborns within 48 hours after childbirth

The continuum of care was considered not complete if the mother missed any of these steps. All the information mentioned in this research is based on self-reports.

#### 2.5.2. Independent Variables

Concerning different variables related to the completion of the maternity continuum of care, we incorporate residence, age, marital status, wantedness of pregnancy, women and their husbands' education status, women and their husbands' employment status, parity, exposure to mass media, and autonomy in health care decision as the factors that might influence the utilization of maternal health care services.

### 2.6. Statistical Analysis

The collected data were coded and entered into EpiData version 3.1. Then, the data were exported to windows of the Statistical Package for the Social Sciences (SPSS) version 20 for data analysis. Bivariable and multivariable logistic regression analyses were used to determine the association of each independent variable with the dependent variable, and those variables with *P* value less than or equal to 0.05 in multivariable analysis were considered significant. A multi-colinearity test was checked by using the standard error. The goodness of fit was tested by the Hosmer-Lemeshow statistic (0.97).

## 3. Result

### 3.1. Sociodemographic Characteristics

Out of the total 651 study participants, 621 of them were included in the final analysis giving a response rate of 95.4%. The mean age of women was 30.85 (SD ±6.56) years. The majority of the respondents (352 (56.7%)) were between 25 and 35 years old, followed by an age group > 35 (159 (25.6%)). Two hundred twenty-seven (36.6%) were housewives, and 225 (36.2%) were farmers. The largest portion of the participants were Orthodox in religion (473 (76.2%)), 513 (82.6) were currently married, and 455 (73.3%) were rural resident constituents (Table 1).

### 3.2. Sociocultural and Husband-Related Characteristics

The findings highlight that around 46.1% of women had access to mass media, 77.1% had autonomy in health care decision-making, and 79.4% of women report that their pregnancy was wanted. Concerning the educational status of the women's husbands, the majority (27.7%) had no formal education, but they can read and write, and regarding occupation of the women's husbands, the majority were farmer (56.8%) (Table 2).

### 3.3. Content, Timing, and Place of Care Received in the Continuum of Care

Among women who received antenatal care, 61.1 percent started care in the first trimester and 22.8 percent in the second trimester. Among women who received antenatal care, 98.5 percent got advice about danger signs during pregnancy. 96.3 percent of women had their blood pressure measured during their antenatal care visit. From a total of women under the study, 15% delivered by caesarian section. Among women who received postnatal care, 99.1 percent and 97.5 percent were counseled about family planning and breastfeeding, respectively (Table 3).

### 3.4. Place of Delivery

**Figure 1:**
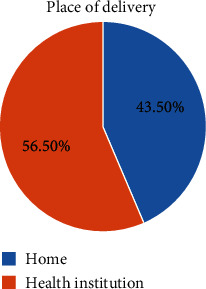
Place of delivery among women who gave birth in the last one year in Enemay District, Northwest Ethiopia, 2019.

In this study, around 56.5 percent of women gave birth in the health institution and 43.5 percent of the study participants gave birth at home (Figure 1).

### 3.5. Mode of Delivery

**Figure 2:**
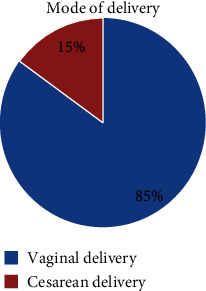
Mode of delivery among women who gave birth in the last one year in Enemay District, Northwest Ethiopia, 2019.

Around eighty-five percent of women in this study delivered vaginally, and fifteen percent delivered with a caesarean section. (Figure 2).

### 3.6. The Continuum of Care

**Figure 3:**
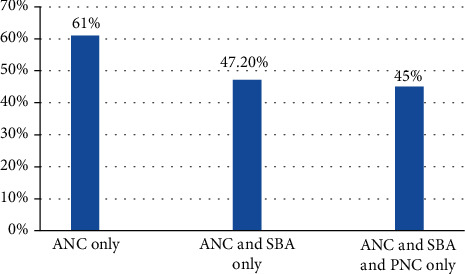
Continuum of maternal health care services among women who gave birth in the last one year in Enemay District, Northwest Ethiopia, 2019.

Around 61% of women received antenatal care, and 13.7% did not continue on the pathway to receive skilled birth attendance. Only 47.2% who received antenatal care were attended by a skilled health provider at delivery. After delivery, 2.2% women did not go on to receive postnatal care (Figure 3).

### 3.7. Completion of the Maternity Continuum of Care

**Figure 4:**
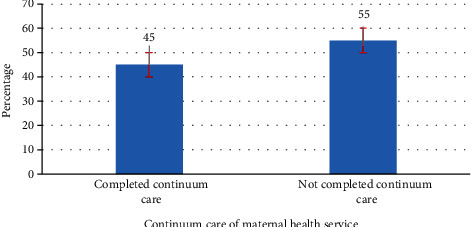
Completion of the maternity continuum of care among women who gave birth in the last one year in Enemay District, Northwest Ethiopia, 2019.

In this study, 45% (95% CI: 40.9%, 48.8%) of women had completed the continuum of care and 55% women did not have the full range of the continuum of care (Figure 4).

### 3.8. Factors Associated with the Completion of the Maternity Continuum of Care

The crude analysis result revealed that some factors such as age group, educational status, residence, exposure to media, autonomy to health care decision-making, parity, wantedness of pregnancy, husband's education, and husband's occupation were associated with the completion of the continuum of care. But in the multivariable analysis, only women's educational status, autonomy to health care decision-making, exposure to media, wantedness of pregnancy, and parity had maintained their statistical association with the completion of the continuum of care.

Women with secondary education were 6 times more likely to complete the continuum of care (AOR = 6, 95% CI: 2.26, 16.6) compared to those women who cannot read and write. Women whose occupation is farming were 82 percent less likely to complete the continuum of care compared to housewives (AOR = 0.18, 95% CI: 0.1, 0.32), and women who have the autonomy to health care decision-making were 4 times more likely to complete the continuum of care (AOR = 4, 95% CI: 2.26, 7.2) compared to those who have no autonomy for health care decision-making.

Women who have exposure to media were 2 times more likely to complete the continuum of care (AOR = 1.97, 95% CI: 1.2, 3.27) compared to those women who have no exposure to media. Women with wanted pregnancy were 3.4 times more likely to complete the continuum of care (AOR = 3.33, 95% CI: 1.87, 5.9) compared to those women whose pregnancy was unwanted, and para five and above women were 2.9 times more likely to complete the continuum of care (AOR = 2.85, 95% CI: 1.28, 6.3) compared to para 1-2 women. In addition, women whose husband is employed are 5 times more likely to complete the continuum of care (AOR = 4.97, 95% CI: 1.16, 21.2) compared to women whose husband is a merchant (Table 4).

## 4. Discussion

In this study, the overall completion of the maternity continuum of care was 45% (95% CI: 40.9%). About 61 percent of women received at least one antenatal care, but only 45% completed the continuum of care, receiving all three types of maternity services, antenatal care during pregnancy, skilled birth attendance at delivery, and postnatal care within 48 hours after delivery. This indicates that about 14% of women drop out from the pathway of the maternity continuum of care before reaching postnatal care. Behind the failure of seeking a complete maternity continuum of care is the fact that after receiving ANC, the majority of the women dropped out from the pathway of the continuum of care. This finding suggests that more dropouts occurred between pregnancy and delivery than between delivery and the PNC period, which is similar to studies conducted in Pakistan [15]. The possible reasons for dropout might be lack of family support and poor counseling during antenatal care services. The other possible reasons for dropout might be the unpredictable onset of labor and difference in sociocultural beliefs between the health care provider and the community.

The magnitude of completion of maternity continuum care in this study was 45% which is in line with study done in Nepal (46%) [3]. The finding was lower than that in the study conducted in Cambodia (60%) [16] and Egypt (51%) [14]. This discrepancy might be due to the difference in health care coverage and difference in the educational status of respondents, since education is one of the strong predictors for the completion of the continuum of care. In this study, the proportion of educated women is 44.6% which is lower than those of Cambodia (82.5%) and Egypt (52.8%). But it was higher than those in studies done in Pakistan (27%) [15], Cambodia (5%) [17], South Asia and sub-Saharan Africa (16.9%) [11], and Nigeria (29%) [18]. This discrepancy might be due to the difference in measurement and variations of the study period and due to the difference in the accessibility of services.

This study had identified a number of important factors that were associated with the completion of the continuum of care for maternal health among women in Enemay District. The multivariate regression analysis shows that education, occupation, autonomy to health care decision-making, wantedness of pregnancy, exposure to mass media, and husband's occupation are the most significant predictive factors for the completion of the continuum of care.

Women with secondary education were more likely to complete the continuum of care compared to those women who cannot read and write. This finding is in line with studies conducted in Pakistan [15], Nepal [14], and Cambodia [16]. The possible reason might be that educated women may have better health knowledge about the importance of receiving maternity care during pregnancy, delivery, and the postnatal period. The other possible reason might be that education is likely to enhance female autonomy and help women to develop greater confidence and capability to make decisions about their own health and educated women may have a good chance to approach the written information about maternal health service.

Having the autonomy to health care decision-making was statistically and positively associated with the completion of the maternity continuum of care. This study is in line with studies done in Pakistan [15] and South Asia and sub-Saharan Africa [11]. This might be due to the fact that women who had autonomy to health care decision-making might have freedom of movement, might not have financial problems, and can go and receive the care by their own selves. Additionally, autonomy may also be associated with other variables like education of women and urban residence, both of which are factors that increase the likelihood of the use of maternal health services.

Completion of the maternity continuum of care was better observed on those women with wanted pregnancy which is similar to the study done in Ghana [19]. The possible reason might be that women with wanted pregnancy are careful to their pregnancy, likely to develop better motivation, and prepared emotionally and financially for the demand of pregnancy and childbearing compared to women with unwanted pregnancy.

Para five and above women were at higher odds of completing the continuum of care compared to para 1-2 women. This study is contrary to the study done in Pakistan [15]. The possible reason for this discrepancy might be that women with higher parity might have better information about the advantage of receiving maternal health service and also women with higher parity may have faced different complications before and have better awareness about the importance of utilizing all the maternal health services. Another possible reason might be that women with higher parity might have frequent contact with health providers in their previous pregnancy and might have gotten educational messages and counseling from health workers before.

Exposure to media was also associated with the completion of the maternity continuum of care. This study is in agreement with studies conducted in Nepal [3], Egypt [14], and Pakistan [15]. This might be that media is one of the means of access to resources for awareness and knowledge, so women who had exposure to media might have gotten information about the importance of receiving maternal health services and also they might have gotten different educational messages regarding maternal health service.

Women whose occupation was farming were less likely to complete the continuum of care compared to housewives. This might be due to the fact that farmers might have lack of time to go to the health institution and most of the farmers live in a rural area where they might have problem accessing health services. The other possible reason might be that farmers might have less information about the advantage of utilizing maternal health service. A husband's employment status was also significantly associated with the completion of the maternity continuum of care. This study is in line with the study done in Egypt [14]. The possible reason might be that women with employed husbands might not have any financial problems and employed husbands might have better information about maternal health service and they might encourage their wives to use the service.

### 4.1. Limitation of the Study

Inferring casual association is difficult due to the cross-sectional nature of the study. It is also difficult to measure the quality of service that women got during their antenatal care, delivery, and postnatal period. In addition, information in the survey is based on self-reports, so there may be social desirable bias and recall bias.

## 5. Conclusion

Less than half of the study participants complete the maternity continuum of care. Women's educational level and occupation, autonomy to health care decision-making, wantedness of pregnancy, exposure to media, parity/number of children, and husband's occupation were significantly associated with the completion of the maternity continuum of care.

## Figures and Tables

**Table 1:** Distribution of the study participants by their sociodemographic characteristics in Enemay District, Northwest Ethiopia (*n* = 621).

Variable	Category	Frequency	Percentage
Respondents' age group	15-24	110	17.7
	25-35	352	56.7
	>35	159	25.6

Respondents' religion	Orthodox	473	76.2
	Others	148	23.8

Respondents' marital status	Single/widowed/divorced	108	17.4
	Married	513	82.6

Respondents' educational status	Cannot read and write	219	35.3
	Able to read and write	125	20.1
	Primary	163	26.2
	Secondary	68	11.0
	College and above	46	7.4

Respondents' occupation	Housewife	228	36.7
	Merchant	127	20.5
	Farmer	226	36.4
	Employed	40	6.4

Parity/number of children	1-2	298	48.0
	3-4	237	38.2
	≥5	86	13.8

Respondents' residence	Rural	455	73.3
	Urban	166	26.7

Others (religion): Muslim = 22.4% and Protestant = 1.4%.

**Table 2:** Sociocultural and husband-related characteristics of women who gave birth in the last one year in Enemay District, Northwest Ethiopia, 2019 (*n* = 621).

Variables	Category	Frequency	Number
Autonomy to health care decision-making	Yes	479	77.1
	No	142	22.9

Respondents' exposure to media	Yes	286	46.1
	No	335	53.9

Wantedness of pregnancy	Yes	493	79.4
	No	128	20.6

Husband's educational status	Cannot read and write	165	26.6
	Able to read and write	172	27.7
	Primary	117	18.8
	Secondary	124	20
	College and above	43	6.9

Husband's occupation	Merchant	213	34.3
	Farmer	353	56.8
	Employed	55	8.9

**Table 3:** Percent distribution of women by content, timing, and place of care received in the continuum of care in Enemay District, Northwest Ethiopia, 2019.

Antenatal care			
Have ANC visit (*n* = 378)		Number	Percent
Timing of the first ANC visit			
First trimester		231	61.1
Second trimester		86	22.8
Third trimester		16	4.2
Do not know		45	11.9
Place of ANC (378)	Public health institution	377	99.7
	Private health institution	21	5.6
Blood pressure measured during ANC	Yes	364	96.3
	No	14	3.7
Blood sample taken during ANC	Yes	361	95.5
	No	17	4.5
Urine sample taken during ANC	Yes	347	91.8
	No	31	8.2
Got advise/counsel during ANC (*n* = 378)	Yes	334	88.4
	No	44	11.6
Got advise about danger signs during ANC	Yes	329	98.5
	No	5	1.5
Got advise about nutrition during ANC	Yes	298	89.2
	No	36	10.8
Got advise about birth and emergency plan	Yes	290	86.8
	No	44	13.2
Take iron folic acid during ANC	Yes	182	48.1
	No	196	51.9
Take tetanus toxoid vaccine during ANC	Yes	165	43.7
	No	213	56.3
Delivery			
Types of delivery (*n* = 621)	Vaginal delivery	528	85
	Caesarian delivery	93	15
Place of delivery (*n* = 621)	Health institution	351	56.5
	Home	270	43.5
Postnatal care			
Place of PNC (415)	Public health institution	415	100
	Private health institution	17	4.1
Got advise during PNC (*n* = 319)			
Got advise about birth spacing and family planning	Yes	316	99.1
	No	3	0.9
Got advise about breastfeeding	Yes	311	97.5
	No	8	2.5
Got advise about hygiene	Yes	240	75.2
	No	79	24.8

**Table 4:** Factors associated with the completion of the maternity continuum of care among mothers who gave birth in the last one year in Enemay District, Northwest Ethiopia, 2019 (*n* = 621).

Variable	Category	Completion		COR (95% CI)	AOR (95% CI)
		Yes	No		
Age group	15-24	60 (54.5%)	50 (45.5%)	2.15 (1.30, 3.53)	1.6 (0.73, 3.5)
	25-35	162 (46%)	190 (54%)	1.53 (1.04, 2.22)	1.17 (0.66, 2.07)
	>35	57 (35.8%)	102 (64.2%)	1	

Marital status	Married	238 (46.4%)	275 (53.6%)	1	—
	Single/divorced	41 (38%)	67 (62%)	0.70 (0.46, 1.08)	

Educational status	Cannot read and write	66 (30.1%)	153 (69.9%)	1	1
	Can read and write	36 (38.8%)	89 (71.2%)	0.94 (0.58, 1.5)	0.93 (0.48, 1.8)
	Primary	82 (50.3%)	81 (49.7%)	2.25 (1.54, 3.5)	1.9 (0.93, 3.94)
	Secondary	56 (82.4%)	12 (17.6%)	10.8 (5.44, 21.5)	6 (2.26, 16.6)^∗∗∗∗^
	College and above	39 (84.8%)	7 (15.2%)	12.9 (5.49, 30.3)	0.8 (0.137, 4.78)

Occupation	Housewife	130 (57%)	98 (43%)	1	1
	Merchant	72 (56.7%)	55 (43.3%)	0.98 (0.64, 1.5)	0.55 (0.28-1.07)
	Farmer	39 (17.3%)	187 (82.7)	0.16 (0.10, 0.2)	0.18 (0.1, 0.32)^∗∗∗^
	Employed	38 (95%)	2 (5%)	14.3 (3.37, 60.8)	8.8 (0.42, 74.6)

Parity	1-2	148 (50%)	148 (50%)	1	1
	3-4	95 (40.1%)	142 (59.9%)	0.68 (0.48, 0.95)	1.6 (0.96, 2.77)
	≥5	36 (41.9%)	50 (58.1%)	0.73 (0.449, 1.18)	2.85 (1.28, 6.3)^∗∗^

Residence	Rural	160 (35.2%)	295 (64.8%)	1	1
	Urban	119 (71.7%)	47 (28.3)	4.67 (3.165, 6.89)	1.28 (0.69, 2.36)

Autonomy to health care	Yes	257 (53.7%)	222 (46.3%)	6.3 (3.87, 10.29)	4.0 (2.26, 7.2)^∗∗∗∗^
	No	22 (15.5%)	120 (80.5%)	1	1

Media exposure	Yes	182 (63.3%)	104 (36.6%)	4.43 (3.15, 6.21)	1.97 (1.2, 3.27)^∗∗∗^
	No	97 (29%)	238 (71%)	1	1

Pregnancy wantedness	Yes	250 (50.5%)	243 (49.3%)	3.51 (2.24, 5.5)	3.3 (1.87, 5.9)^∗∗∗∗^
	No	29 (22.7%)	99 (77.3%)	1	1

Husband's education	Cannot read and write	57 (34.5%)	108 (65.5%)	1	1
	Can read and write	56 (32.6%)	116 (67.4%)	0.91 (0.58, 1.49)	0.66 (0.35, 1.25)
	Primary	50 (42.7%)	67 (57.3%)	1.41 (0.87, 2.3)	0.89 (0.42, 1.9)
	Secondary	78 (62.9%)	46 (37.1%)	3.21 (1.98, 5.2)	0.6 (0.26, 1.4)
	College and above	38 (88.4%)	5 (11.6%)	14 (5.37, 38.6)	0.48 (0.85, 2.79)

Husband's occupation	Merchant	126 (59.2%)	87 (40.8%)	1	1
	Farmer	104 (39.5%)	249 (70.5%)	0.29 (0.202, 0.4)	1.34 (0.65, 2.8)
	Employer	49 (89.1%)	6 (10.9%)	5.64 (2.31, 13.7)	4.97 (1.16, 21.2)^∗^

^∗^Significant with *P* = 0.03, ^∗∗^significant with *P* = 0.01, ^∗∗∗^significant with *P* = 0.008, and ^∗∗∗∗^significant with *P* = 0.0001.

## Data Availability

The data used to support the findings of this study are available from the corresponding author upon request.

## Abbreviations

ANC: Antenatal care
AOR: Adjusted odds ratio
BSc: Bachelor of Science
CI: Confidence interval
COC: Continuum of care
DHS: Demographic and Health Survey
EDHS: Ethiopian Demographic and Health Survey
ETB: Ethiopian birr
GDP: Gross domestic production
PDHS: Pakistan Demographic and Health Survey
PI: Principal investigator
PNC: Postnatal care
SKB: Skilled birth attendant
SPSS: Statistical Package for the Social Sciences
WHO: World Health Organization
US$: United States dollar.
